# An evaluation of the telehealth facilitation of diabetes and cardiovascular care in remote Australian Indigenous communities: - protocol for the telehealth eye and associated medical services network [TEAMSnet] project, a pre-post study design

**DOI:** 10.1186/s12913-016-1967-4

**Published:** 2017-01-05

**Authors:** Laima Brazionis, Alicia Jenkins, Anthony Keech, Chris Ryan, Sven-Erik Bursell, On behalf of the TEAMSnet Study Group

**Affiliations:** 1 Department of Medicine, The University of Melbourne, Melbourne, VIC Australia; 2 Sansom Institute, University of South Australia, Adelaide, SA Australia; 3 Department of Nutrition and Dietetics, La Trobe University, Melbourne, VIC Australia; 4 NHMRC Clinical Trials Centre, University of Sydney, Sydney, NSW Australia

**Keywords:** Indigenous, Telehealth, Type 2 diabetes, Cardiovascular disease, Retinopathy, Nephropathy, Retinal imaging, Electronic decision support, Mobile tablet, Diabetes education

## Abstract

**Background:**

Despite substantial investment in detection, early intervention and evidence-based treatments, current management strategies for diabetes-associated retinopathy and cardiovascular disease are largely based on real-time and face-to-face approaches. There are limited data re telehealth facilitation in type 2 diabetes management. Therefore, we aim to investigate efficacy of telehealth facilitation of diabetes and cardiovascular disease care in high-risk vulnerable Aboriginal and Torres Strait Islanders in remote/very remote Australia.

**Methods:**

Using a pre-post intervention design, 600 Indigenous Australians with type 2 diabetes will be recruited from three primary-care health-services in the Northern Territory. Diabetes status will be based on clinical records. There will be four technological interventions: 1. Baseline retinal imaging [as a real-time patient education/engagement tool and telehealth screening strategy]. 2. A lifestyle survey tool administered at ≈ 6-months. 3. At ≈ 6— and 18-months, an electronic cardiovascular disease and diabetes decision-support tool based on current guidelines in the Standard Treatment Manual of the Central Australian Rural Practitioner’s Association to generate clinical recommendations. 4. Mobile tablet technology developed to enhance participant engagement in self-management. Data will include: Pre-intervention clinical and encounter-history data, baseline retinopathy status, decision-support and survey data/opportunistic mobile tablet encounter data. The primary outcome is increased participant adherence to clinical appointments, a marker of engagement and self-management. A cost-benefit analysis will be performed.

**Discussion:**

Remoteness is a major barrier to provision and uptake of best-practice chronic disease management. Telehealth, beyond videoconferencing of consultations, could facilitate evidence-based management of diabetes and cardiovascular disease in Indigenous Australians and serve as a model for other conditions.

**Trial registration:**

Australia and New Zealand Clinical Trials Register (ANZCTR): ACTRN 12616000370404 was retrospectively registered on 22/03/2016

## Background

Diabetes and cardiovascular disease (CVD) are leading causes of personal and healthcare system disease burden, globally [1], and in Australia [2]. It is anticipated that this burden will disproportionately affect vulnerable populations, such as those in low-income countries and Indigenous populations [3], particularly in remote settings.

A wide range of interventions aimed at improving chronic disease outcomes and reducing care costs has been tried. These interventions mostly fall into one of two categories. One aspect is quality-improvement that focuses on service delivery in primary care and support services for patient self-management. The other aspect is patient-based disease-management. Evidence suggests the interventions that not only target disease management, but also aim to improve care-delivery design may be more effective than those that target disease management alone [4, 5]. Targeted e-health applications that facilitate both service delivery and disease management have potential to meet such an objective. To date, clinical e-health applications mainly relate to the use of electronic health record systems (often alongside older paper-based systems), telecommunication of medical reports and videoconferencing consultations between a clinician and patient.

Telehealth is, in the context of the present study, that area of e-health that centres on the provision of health-related services and information using telecommunication-based technologies. Telehealth in the clinical setting is in its infancy in Australia [6], where the use of this term has been largely limited to one Federal government funded health-related service — that of a videoconferencing consultation between a specialist clinician and patient, subject to specific criteria [7]. By contrast, global uptake of a broad spectrum of e-health technologies, particularly mobile-health, is high [8].

Telehealth facilitation of service delivery and disease management has the potential to improve health outcomes for people with diabetes and to improve the quality of service delivery in the primary care setting. Currently, there are inadequate data to support the widespread clinical uptake of telehealth technologies. Therefore, the ‘Telehealth Eye and Associated Medical Services Network’ [TEAMSnet] study aims to address the knowledge gaps in three areas — telehealth facilitation of 1. diabetic retinopathy screening 2. evidence-based diabetes and CVD management (via decision support and survey tools) and 3. patient self-management strategies.

This paper presents the protocol for the TEAMSnet study. This technology-based pre-post intervention study aims to describe the baseline characteristics of the participants with diabetes and clinical workflows in partner health service and to investigate the impact of telehealth facilitation in the management of CVD, diabetes and diabetic retinopathy. We hypothesise that telehealth facilitation of diabetes management will lead to improvements in patient engagement [attendance rates and survey participation] and clinical outcomes, such as blood pressure and metabolic control.

## Methods/Design

### Study design

This is a pre-post study of technology-facilitated management of diabetes, CVD, and diabetic retinopathy. We plan to enrol 600 adults (age ≥18 years) with clinically-diagnosed diabetes from three remote Northern Territory Aboriginal-controlled health services — Central Australian Aboriginal Congress [CAAC] in Alice Springs, Miwatj Health Aboriginal Corporation [Miwatj] that incorporates the four clinics at Nhulunbuy, Galiwin’ku/Elcho Island, Yirrkala, Gunyangara/Ski Beach in East Arnhem Land and Wurli-Wurlinjang Health Service [Wurli] in Katherine. Recruitment and intervention are anticipated to occur over a 3-year period. Participants will receive retinal imaging, diabetic retinopathy screening, including diabetes education, at baseline, followed by lifestyle assessment six months later, including self-management support. An electronic CVD and diabetes decision support tool that includes an Indigenous-specific CVD risk calculator, based on best-practice as generally defined by guidelines in the current Standard Treatment Manual of the Central Australian Rural Practitioner’s Association [CARPA], will be developed in conjunction with a clinical working group and used to generate a clinical recommendations report to assist clinicians with evidence-based management of CVD, diabetes and diabetic retinopathy approximately 6–18 months after baseline. Mobile tablet technology based on clinical input and feedback will be developed to support clinical staff and participants with engagement in diabetes management and self-management, respectively.

### Study aims and objectives

The overarching aim of the TEAMSnet project is to improve health care, and therefore health outcomes, for Indigenous Australians in under-resourced communities through the integration of existing electronic health record systems, specifically Communicare™ (Healthconnex, Perth, Australia), with the customisable, web-based health information technology called Chronic Disease Management Platform [CDMP], an existing, licence-free platform that thereby facilitates sustainability. A secondary aim is to evaluate the cost-efficacy of the intervention. There are three main objectives under the broad aim.

1. *Telehealth facilitation of clinical management* via *CVD and diabetes electronic decision support and remote image-based diabetic retinopathy assessment*
  Our first objective is to make it easier for treating doctors to adhere to relevant evidence-based guidelines, specifically the current Standard Treatment Manual of the Central Australian Rural Practitioner’s Association (CARPA) [9] and the 2008 NHMRC Guidelines for the Management of Diabetic Retinopathy [10]*,* prepared by the Australian Diabetes Society. To this end, (1) CARPA guidelines are being encoded into a CARPA_CVD and diabetes electronic decision support application [EDS] that sits within CDMP. In addition, evidence-based diabetic retinopathy management has been made easier for primary care practitioners via (2) the development of a second application, Global Retinal Imaging [GRI], that interfaces with the widely–used Centervue DRS and Canon CR-2 non-mydriatic retinal cameras and stores the captured digital retinal and external eye images. These imaging, grading and reporting data are accessible from within CDMP to both certified retinopathy graders [for grading diabetic retinopathy level and generating retinopathy reports based on 2008 Australian NHMRC diabetic retinopathy management guidelines] and to authorized medical and eyecare practitioners [to view and compare ocular images and retinal status over time, thereby facilitating continuity of care].
2. *CDMP-based facilitation of shared clinical information, such as login access to CDMP-stored retinal images for ophthalmologists and optometrists*
  Our second objective is to improve the timely exchange of relevant health information and the communication between a patient’s health care providers. CDMP facilitates this through use of a unique practitioner access code that enables a specialist medical practitioner, such as an optometrist or ophthalmologist, to view only relevant patient data, including reports.
3. *Improve engagement of patients in self-care, particularly adherence to clinical visits*
  Our third objective is to improve engagement of patients in self-care. GRI retinal imaging will provide both a powerful education and retinopathy assessment opportunity. In addition, a third technology, (3) a custom-built e-mobile health device in the form of a tablet with viewable clinical data, GRI images and educational content, developed with clinical and Aboriginal Health Worker input, will facilitate health worker communication with patients between medical consultations and is aimed at improving relationships and engaging patients in self-care. A fourth application, (4) a suite of lifestyle surveys and a related reporting template based on CARPA recommendations for smoking, nutrition, alcohol, physical activity and emotional well-being [SNAPE] has been developed and is accessible via CDMP in a clinical setting or via the mobile tablet application anywhere. This is designed to be both a patient engagement and clinical management tool that facilitates the measurement, goal-setting and monitoring of important lifestyle risk factors for CVD, diabetes and diabetic retinopathy. All three telehealth reports, EDS, GRI and SNAPE, will be accessible via either the EHR [Communicare] or CDMP.

Apart from the development and implementation of the four technologies, the primary outcome includes improved adherence to clinical care, such as eyecare visits. Secondary outcomes include reduction in hospitalizations and cost-efficacy of the suite of technologies, both individually and collectively.

### Site recruitment

An invitation was circulated to Aboriginal-Controlled Community Health Organizations [ACCHO] in the Northern Territory to attend an information workshop at the Alice Springs Hospital. Following this, an expression of interest was sought from attendees. A discussion paper was circulated for internal discussions within the health service to the three health services that formally expressed an interest in the project. Once health service board approval had been obtained, a formal letter of support from the health service for the TEAMSnet study was sought and Memoranda of Understanding and service agreements were executed between partner sites and the administering institutions, the Universities of Melbourne and Sydney. Ethics applications were submitted by investigators to the Central Australian and Menzies School of Health Research Human Research Ethics Committees. Recruitment commenced once written ethics approval had been obtained.

### Participant eligibility

#### Inclusion criteria

Participants must be clients of the partner health services who are aged 18 or over; can provide informed consent; can successfully undergo retinal imaging; are willing to undertake surveys and provide relevant clinical information; and are willing to consult their doctor on at least two occasions over the following 18 months.

#### Exclusion criteria

Clients of the partner health services are ineligible if they been clients for less than six months prior to enrollment or have had diabetic retinopathy treatment.

### Sample recruitment

Both community-based and direct recruitment strategies are being used to recruit study participants. These include; flyers in medical waiting and consulting rooms, and contact with potential referral sources, for example, general practitioners, chronic care team members — podiatrist, dietitian, cardiac nurse, renal nurse, diabetes educator, text and letter invitations.

Prospective participants express their interest to the study co-ordinator at a face-to face meeting during which the individual is screened. If the individual meets the inclusion criteria, they are consented to the study (consent form signed after patient information sheet discussed) and diabetic retinopathy screening is performed (baseline visit) in order to reduce the time burden on participants and improve compliance with the study protocol.

### Study procedure

#### TEAMSnet telehealth protocols

**Fig. 1:**
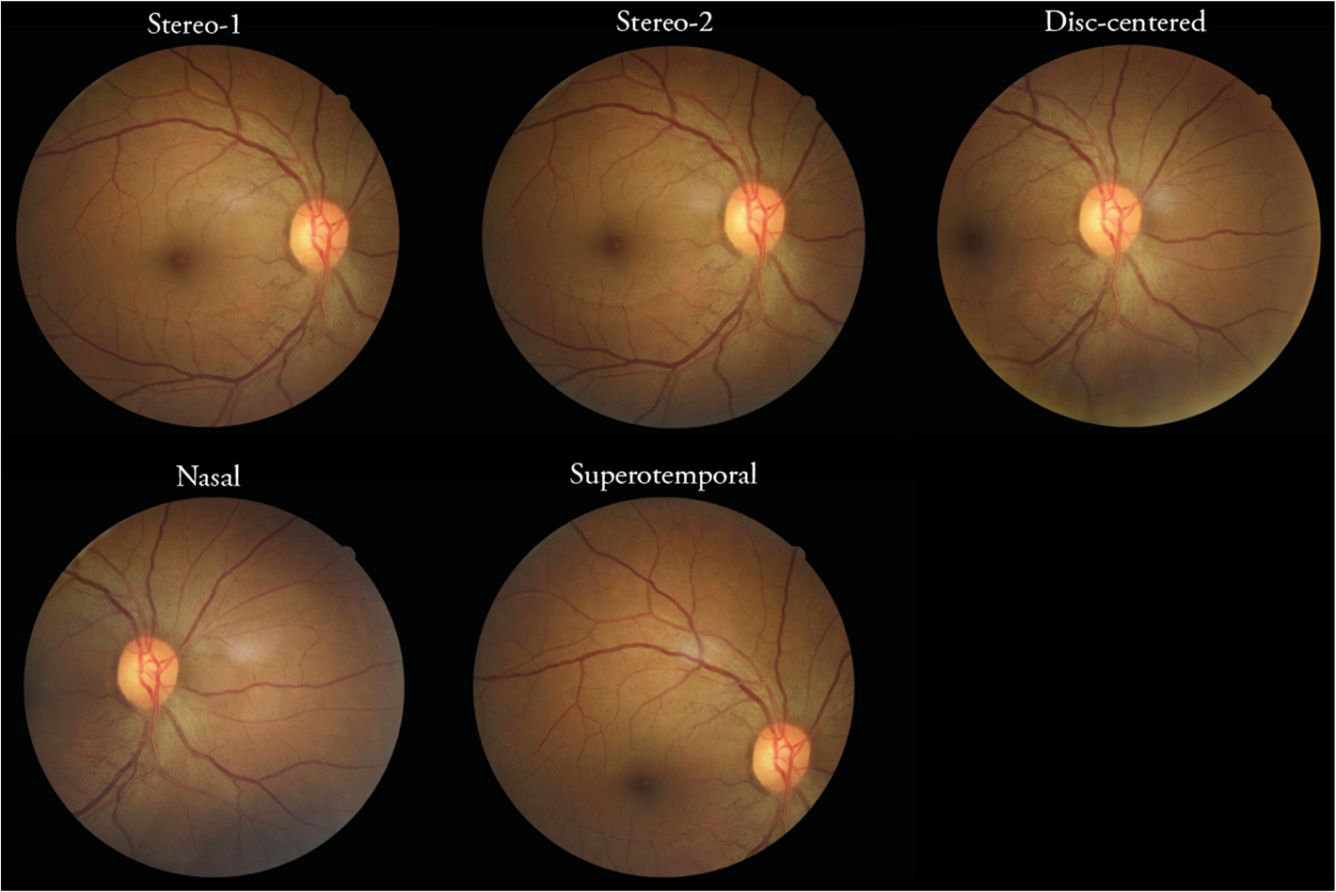
Diabetic Retinopathy Screening imaging fields
  Key elements of the diabetic retinopathy grading protocol are as follows —
  1. Grading of retinopathy and vessel calibre measurements are being undertaken by Centre for Eye Research Australia [CERA]-certified graders at the Royal Victorian Eye and Ear Hospital, Australia [RVEEH] grading centre, with ophthalmological adjudication as required.
  2. Images are first read for presence and extent of retinal pathologic lesions (haemorrhages and microaneurysms, venous beading etc.) i.e. number of specific lesions per retinal quadrant per image per eye are documented electronically according to the Early Treatment Diabetic Retinopathy Study standardised grading protocol. A grading algorithm based on the Early Treatment Diabetic Retinopathy Study [ETDRS] classification of diabetic retinopathy analyses the electronically-entered retinal lesion data automatically calculates a preliminary clinical level of diabetic retinopathy. This initial grading is either accepted or modified by a grader after comparison with the relevant ETDRS standard field.
  3. The referral protocol (Fig. 2) is based on the 2008 NHMRC Guidelines for the Management of Diabetic Retinopathy.
  4. The reporting protocol (Fig. 3) has been developed with a view to highlighting the essential management recommendation in the TEAMSnet retinopathy screening report that is sent to Communicare at the participating site via Argus messaging (a third party vendor providing secure communications between health services) for the primary care health professional to evaluate and action in the context of the participant’s prior ocular history in the EHR [as is the case with pathology reports].
  5. Subsequently, the disc-centred images in a specific format will be used by graders for retinal vessel calibre measurements, which have been shown to predict CVD and retinopathy onset and progression (Fig. 4) [11, 12].

1. Baseline visit — Retinal imaging-based diabetic retinopathy screening
  Imaging of participants by TEAMSnet trained clinic staff aims to provide a valuable patient education and engagement opportunity in addition to facilitating the timely detection of diabetic retinopathy.
  Our diabetic retinopathy screening method is based on a protocol developed in collaboration with the Joslin Diabetes Centre in the USA. Key features are — (1) Digital Imaging and Communications in Medicine (DICOM) compliant images from digital non-mydriatic retinal fundus cameras are cached or transmitted over the internet using Transport Layer Security [TLS] encryption [based on connectivity] via the GRI application to a remote grader. (2) Grading and reporting to Communicare at the originating site is completed within two working days of the grading centre receiving the imaging studies that can be viewed as an active work list on the grader’s computer system. (3) Images can be viewed anytime, anywhere in the web-based CDMP application by clinic-authorized personnel. Optometrists, ophthalmologists, primary care doctors, endocrinologists, nurses, health workers and retinal imagers will be provided with filtered levels of information via a unique access code.
  The main elements of the retinal imaging protocol are —
  1. History — Patient demographic data are entered. Presenting vision is recorded [pinhole if below 6/6 when possible], either unaided or aided by spectacles. If presenting vision is unaided, but patient has distance vision spectacles, then acuity with spectacles is also recorded. Date of last eye exam or any significant ocular history [surgery, trauma] is noted electronically in the GRI intake template text field.
  2. Preparation for imaging — Dilating drops [one each of 1% tropicamide and 2.5% phenylephrine] are instilled, unless contraindicated or patient refuses permission to instil drops, and the patient waits for 15 min or until the pupil diameters are at least 4 mm. We use the waiting time of 15 min to discuss the appearance of a normal retinal and the value of retinal imaging as a window into vascular health. In the absence of pupil dilation and before being imaged the patient requires five minutes [minimum] of dark adaptation in a windowless or pitch black room in order to maximize pupil size.
  3. Imaging sequence — a total of 6 images per eye are acquired, two macula-centred [stereo] fields are imaged first for each eye. The disc-centred field is the third field to be imaged [for vessel calibre and disc assessment]. The nasal and superior-temporal fields for documenting diabetic retinopathy and other findings are the fourth and fifth retinal fields, respectively. The sixth field is the anterior eye/external field for documentation of ocular surface and lid pathology and for future patient identification (Fig. 1).
  4. Camera maintenance — Procedures for camera start up, close down, lens cleaning, storage and syncing with the GRI application have been documented for use by site staff.

**Fig. 2:**
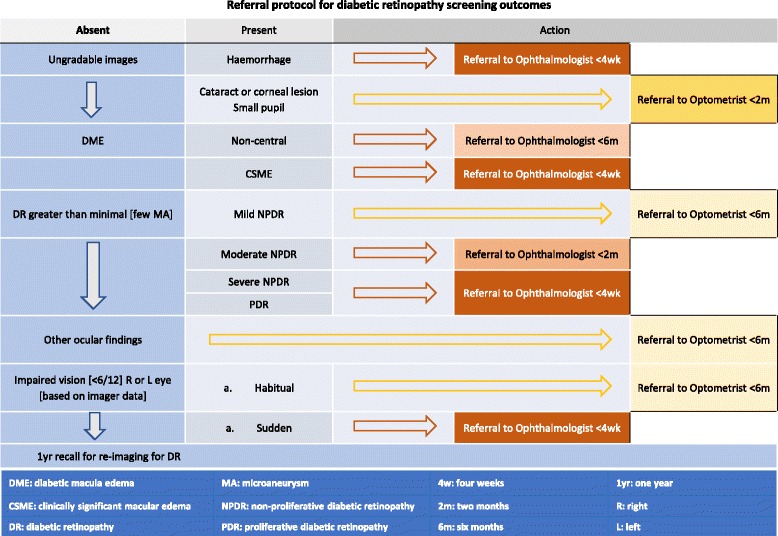
Diabetic Retinopathy Screening referral protocol

**Fig. 3:**
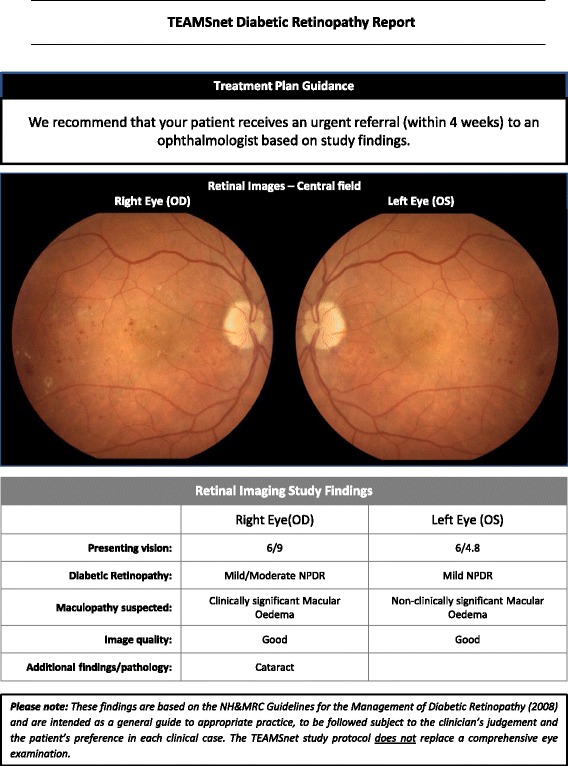
Diabetic Retinopathy Screening reporting protocol

**Fig. 4:**
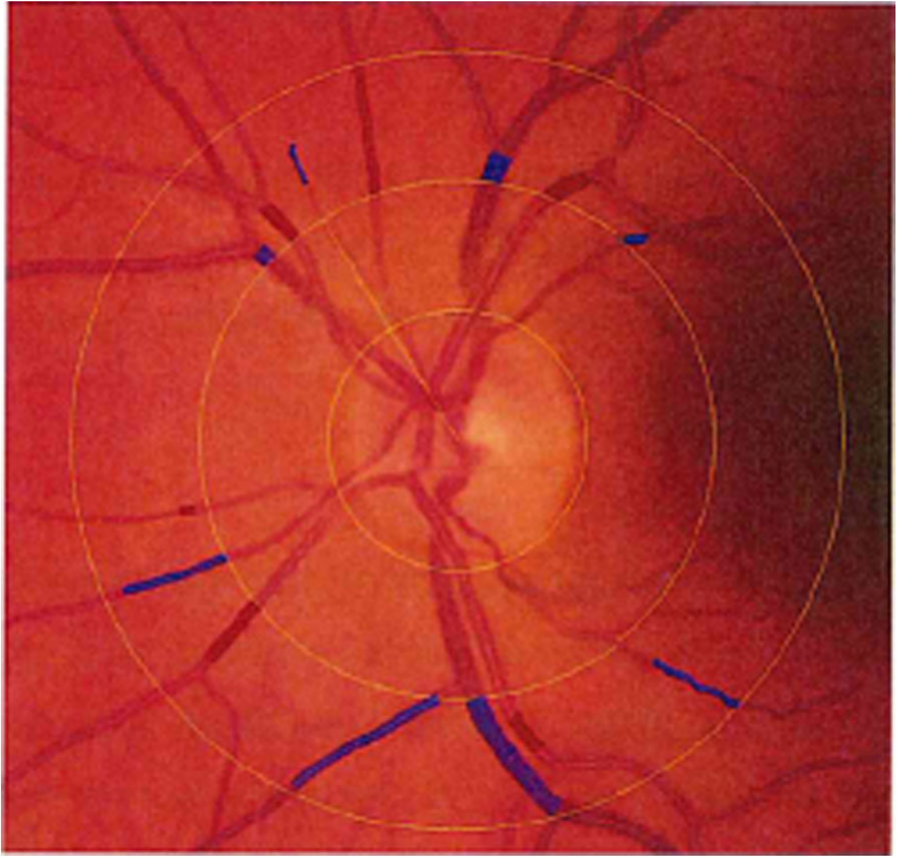
Retinal vessels used for calibre measurement protocol
2. SNAPE lifestyle survey development, administration, reporting and referral protocols
  Validated surveys for smoking, alcohol, physical activity and emotional wellbeing/depression have been codified into a survey tool for online use via CDMP or mobile tablet login. For offline use a print copy of the SNAPE survey reporting template can be used. A relevant and validated nutrition survey is not available so experienced research nutritionists and clinicians have developed a brief diet quality questionnaire adapted from recent studies in Indigenous health.
  Trained study staff will administer the suite of surveys during the second study visit some 6- months after the imaging visit. The SNAPE report (Fig. 5) can be viewed either in the EHR systems (within 24 h) or immediately via login to CDMP or the mobile tablet. The primary care clinician upon review of the report will generate a referral as indicated. High depression scores are brought to the immediate attention of the participant’s treating clinician. Participants’ comments around goals and barriers/motivators to change, such as food security (access to healthy food), will be documented by the survey administrator.

**Fig. 5:**
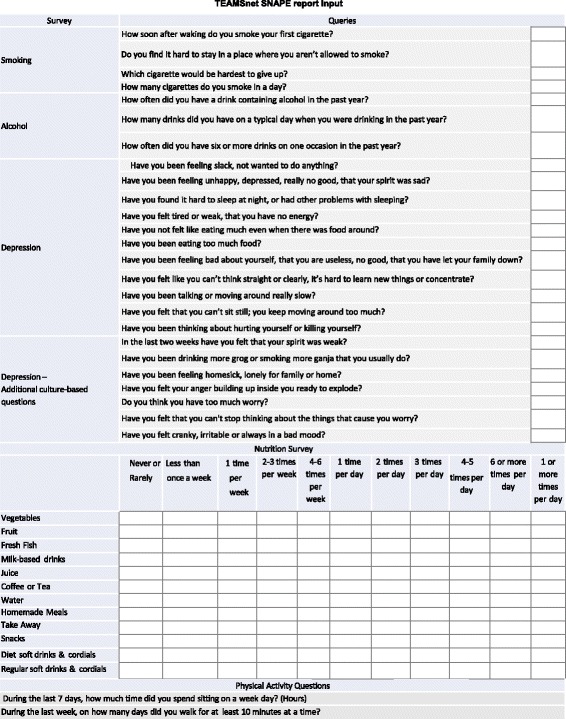
SNAPE lifestyle survey report
3. EDS development, use and reporting protocol
  Once partner sites were identified, we identified the CARPA standard treatment manual as the most appropriate guideline for CVD and diabetes EDS development. As Communicare is the EHR system used by all partner sites, ideally the TEAMSnet EDS application would need to be interfaced with Communicare for automated syncing of as much of the clinical data required by EDS as possible so as to avoid duplication and improve clinical efficiency. The developers of the CARPA standard treatment manual and Communicare were advised of the TEAMSnet project and agreed to support the project informally as advisors and collaborators, particularly in relation to timing of their product updates and releases. In return, TEAMSnet would provide both organisations with potentially useful user feedback during the project.
  TEAMSnet EDS will provide clinicians with three CVD and diabetes management tools.
  1. The **CVD risk calculator and slide rule** that enables baseline measurement and regular monitoring of a participant’s overall CVD risk (Fig. 6).
  2. The **detailed CVD and diabetes risk-analysis tool** that provides a clinician with specific recommendations and optional learning points for best-practice management of a participant’s CVD risk (Fig. 7).
  3. **Active CVD and diabetes decision-support tool** — a proactive clinical process whereby each recommendation is reviewed by the clinician and either accepted or rejected, in which case a textbox appears for the explanation. An EDS report will be generated for each participant as soon as possible after the second clinical visit [at which surveys are administered]. A second and final EDS report will be generated for participants prior to the end of the study (Fig. 8).

**Fig. 6:**
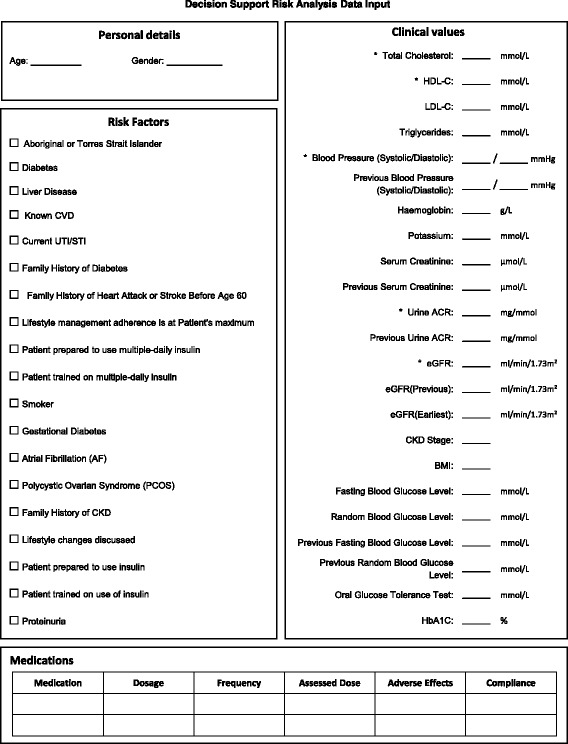
CARPA-designated risk analysis inputs

**Fig. 7:**
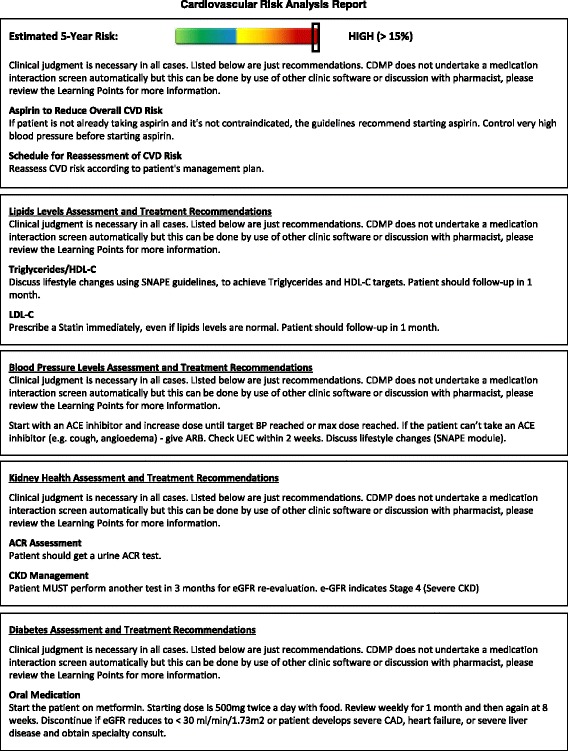
CARPA-based risk analysis report

**Fig. 8:**
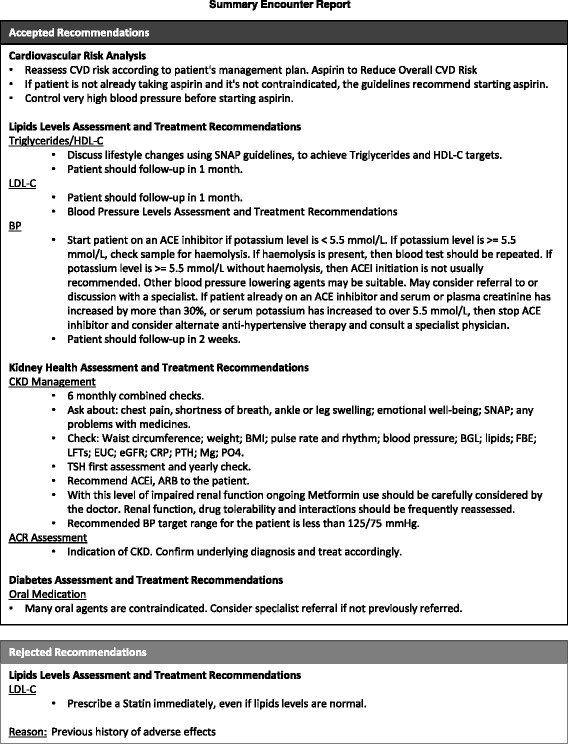
CARPA-based EDS report with accepted and rejected risk analysis recommendations
4. Mobile tablet application
  Mobile tablet development and use will enable display and review of much of the data derived from GRI, SNAPE surveys and EDS applications. Surveys can be administered and an educational resource library can be populated and accessed via the tablet, and this can take place in an out-of-clinic setting, thereby facilitating patient engagement.

### Data collection and outcome measures

Demographic data (including age, gender, postcode, contact details) and self-report measures are obtained from each participant at baseline and/or at the 6-month survey administration visit. Pre- and post-study clinical data such as blood and urine biochemistry, blood pressure and anthropometric measures including height and weight are also collected. Pre- and post-study health care resource utilization is captured through EHR records and includes information on hospitalizations and eye-care consultations.

#### Pre-study data

Site partners will collaborate to audit the Communicare EHR for each participant in order to obtain the required pre-study data i.e. the data that correspond with post-study data relating to CVD, diabetes or diabetic retinopathy risk and outcomes and each participant’s use of health services, such as eye-care consultations and hospitalizations.

### Data management

The study coordinators at respective sites will be responsible for electronic scanning and storage of hard copies of participant consent and report forms for entry into the password protected central database. Hard copies will be kept in a secure cabinet at the respective sites. If requested, participants can access their individual results at study completion.

### Study integrity

Approval to conduct the study was received from Central Australian and Menzies School of Health Research Human Research Ethics Committees in the Northern Territory. Written informed consent will be obtained from all participants. A local translator will assist in consenting patients for whom English is not their first language.

## Sample Size

For a conservative differential in adherence to subsequent care of 15%, and with 80% power, the minimum sample required is 490. Estimates are based on a 25% attrition rate. We will therefore need 600 participants across the three partner sites.

## Data analysis

Investigators and their supporting statistics departments will conduct data analysis. Site differences will be analysed. Analyses will provide important descriptive data, such as prevalence rates and lifestyle data, proportion treated to target for CVD, diabetes and diabetic retinopathy risk factors and predictors of diabetic retinopathy and CVD outcomes. Analysis of covariance (ANCOVA) will be used to compare differences between groups. To assess confounding, covariates will include age, gender, and established and emerging risk factors for CVD, diabetes and diabetic retinopathy, as appropriate. Non-parametric statistics will be used when assumptions for parametric methods are violated. All tests will be conducted using an alpha level of 0.05 and 95% confidence intervals will be reported.

Complete case analysis and, where reasonable, multiple imputation will be employed for the management of missing data. Missing data may occur due to drop-out, missed assessments or item non-response. With knowledge of the missing data mechanism, all available information can be used. One approach will be to use the multilevel model, a full-likelihood method that allows for a response to be missing at random, where missingness depends only on observed data and not on unobserved data. Another strategy involves imputing missing values by multiple imputation procedures.

The proposed economic evaluation aims to include pre- and post-study resource utilization costs for both the costs and consequences and a cost consequence analysis (CCA). Standard unit costs i.e. Australian Medicare Benefits Schedule will be applied to the resource-use data. Costs will be presented in total and disaggregated forms, such as those from different sectors — the health sector, patients and government.

Of the recruitment target of 600 Indigenous Australians with diabetes, to date 576 participants have been recruited and undergone retinal imaging for diabetic retinopathy screening.

## Discussion

It is well-known that a significant number of individuals with diabetes fail to adhere to screening recommendations and/or adequately self-manage diabetes-related risk factors and consequences. Furthermore, primary care service delivery for diabetes and its complications is challenging, even in well-resourced environments with a large proportion of patients at low risk of diabetes complications and related conditions, such as depression. Importantly, the disease burden of diabetes in Australia is greatest amongst Indigenous Australians, particularly in remote settings.

Telehealth facilitation of service delivery and diabetes management has potential benefits. To date, there are insufficient data to answer the question ‘Can telehealth applications facilitate best-practice in diabetes and CVD service delivery and disease management in remote Indigenous Australian communities?’ The TEAMSnet study will provide evidence regarding the efficacy and cost-efficacy of telehealth facilitation in the management of diabetes, its complications and related conditions in remote and very remote Indigenous Australian communities. We anticipate benefits in the area of new and modified diabetes management strategies alongside improvements in patient self-management.

This multi-pronged telehealth approach may facilitate the management of diabetes, an increasingly prevalent and complex chronic condition and public health problem. The benefits of telehealth facilitation of retinal screening, lifestyle modification and electronic decision support might then be extended to other complications of diabetes and common co-morbidities.

## Abbreviations

ANCOVA: Analysis of covariance
ANZCTR: Australia and New Zealand Clinical Trials Register
CAAC: Central Australian Aboriginal Congress
CARPA: Central Australian Rural Practitioner’s Association
CDMP: Chronic Disease Management Platform
CERA: Centre for Eye Research Australia
CVD: Cardiovascular disease
DICOM: Digital Imaging and Communications in Medicine
DRS: Diabetic retinopathy screening
EDS: Electronic decision support
EHR: Electronic health record
ETDRS: Early Treatment Diabetic Retinopathy Study
GRI: Global retinal imaging
NHMRC: National Health and Medical Research Council of Australia
RVEEH: Royal Victorian Eye and Ear Hospital, Australia
SNAPE: Smoking, nutrition, alcohol, physical activity and emotional well-being
TEAMSnet: Telehealth Eye and Associated Medical Services Network
